# What aspects of the pandemic had the greatest impact on adolescent mental health: duration of lockdown or subjective experience?

**DOI:** 10.1186/s13034-024-00759-3

**Published:** 2024-06-01

**Authors:** Hiroko Fujimoto, Anita Heywood, Kate Maston, Lyndsay Brown, Alexandra Bartholomew, Aliza Werner-Seidler, Helen Christensen, Philip J. Batterham

**Affiliations:** 1 Black Dog Institute, Sydney, NSW Australia; 2 School of Population Health, University of New South Wales, Sydney, NSW Australia; 3 School of Clinical Medicine, Discipline of Psychiatry and Mental Health, University of New South Wales, Sydney, NSW Australia; 4 School of Psychology, University of New South Wales, Sydney, NSW Australia; 5 Centre for Mental Health Research, The Australian National University, Canberra, ACT Australia

**Keywords:** COVID-19, Lockdowns, Adolescents, Mental health, Wellbeing

## Abstract

**Background:**

The COVID-19 pandemic negatively impacted global mental health, with adolescents experiencing disproportionate effects. Limited research has explored the impact of different pandemic restrictions on adolescent mental health, and only a few studies have examined the longer-term impacts of the pandemic on adolescent mental health. These investigations are crucial for informing public health policies, particularly the integration of mental health care in future public health emergencies.

**Methods:**

This study aimed to investigate the impact of lockdown duration and the impact of adolescents’ subjective experiences of the pandemic on their wellbeing, internalising symptoms, and externalising symptoms. Australian adolescents (*N* = 1,001, mean age = 14.2 years) completed a baseline survey in 2021, shortly after pandemic lockdowns were lifted (Time 1), and a follow-up survey approximately 12 months later (Time 2). Predictors of interest were the total duration of COVID-19 lockdowns across 2020–2021, and adolescents’ subjective experiences of the pandemic on their social connections, learning, technology use and family relationships. A range of covariates were included in analyses to examine subgroup differences.

**Results:**

Linear mixed-effects models indicated that total duration of the lockdown was not associated with any of the outcomes at Time 1 or Time 2 (all *p*s > 0.017). Negative subjective experience of the pandemic on learning was associated with greater externalising symptoms at both Time 1 (t = 5.17, df = 980, *p* <.001) and Time 2 (t = 2.72, df = 708, *p* =.007). Negative subjective experience of the pandemic on social connection was associated with greater internalising symptoms at Time 2 only (t = 3.20, df = 709, *p* =.001). Negative subjective experience of the pandemic on family relationships or technology use was not associated with any of the outcomes at Time 1 or Time 2 (all *p*s > 0.017).

**Conclusions:**

Adolescents’ negative subjective experience of the pandemic on learning and social connections was associated with greater internalising and externalising symptoms after the lockdown had been lifted. Duration of lockdowns was not associated with any of the primary outcomes. During future public health emergencies, mental health interventions should be tailored to assist adolescents to adapt to new learning environments, and to build and maintain social connections.

**Supplementary Information:**

The online version contains supplementary material available at 10.1186/s13034-024-00759-3.

## Background

The COVID-19 pandemic negatively impacted the mental health of the global population, particularly adolescents [1, 2]. This heightened vulnerability amongst adolescents may be related, at least partly, to their context. That is, to living in an increasingly anxiogenic environment where socioeconomic, environmental, and political factors, including international pandemics, combine to drive the onset and continuity of anxiety [3]. Many of these factors associated with mental health disproportionately affect adolescents [4] and may have been intensified by the pandemic. Another factor that may have increased adolescents’ vulnerability to mental health challenges during the pandemic is their peer-based psychosocial development. The pandemic restrictions, including social distancing, school closures, and stay-at-home orders limited adolescents’ social contact with their peers and may have inadvertently disrupted this psychosocial development [5] which is central to adolescent identity formation [6].

In four years since the initial implementation of the COVID-19 restrictions, there have been several systematic reviews of cross-sectional studies [2, 7, 8] and longitudinal studies [1, 2], all showing the overall negative impact of the pandemic restrictions on adolescents’ mental health. A recent meta-analysis suggests that the global prevalence of mental health symptoms among adolescents doubled from pre-pandemic times to during lockdown when 25% of young people were estimated to experience clinical levels of depression and 20% estimated to experience clinical levels of anxiety [9]. However, it is important to note that most of the data from these studies was collected at the initial phase of the pandemic (up to November 2020), leaving a gap in our understanding of the enduring repercussions of the pandemic, including during the post lockdown period. Existing research exploring longer-term effects has yielded mixed results [1, 2]. Some longitudinal studies investigated young people’s mental health during the transition period from the lockdowns to school re-opening [10–12]. For example, a US study found that depression and anxiety symptoms decreased to pre-pandemic levels after schools re-opened [10], while an Australian study found that when schools re-opened post pandemic, there was a significant increase in depression symptoms and externalising symptoms, and a decrease in mental wellbeing [11]. Another study found a positive association between COVID-19 related stressors during the lockdowns and mental health symptoms six months after school re-opening [12].

These mixed findings could be attributed to heterogeneity across these studies. For example, one major difference is that across countries and regions, experiences of the pandemic, including case counts, stringency and duration of the lockdown restrictions varied significantly. Additionally, heterogeneity arises from the utilisation of different assessment tools and measures for gauging experiences across studies. Numerous studies employed subjective measures to assess the impact of the pandemic, for example, stress, fear, concerns and worry regarding the impact of the pandemic on learning [2, 8, 13–15], social connections and family relationships [2, 14–16]. Although these subjective assessments are valid methods to measure the impact of the pandemic, it is also necessary to complement them with more objective measures of the impact of pandemic restrictions on mental health and wellbeing, especially given that objective aspects of the restrictions could be modified. For example, better knowledge about the association between duration of lockdowns and mental health can quantify the toll that such public health measures exact on mental health and wellbeing. This might also indicate whether there was a threshold level at which the lockdown began to impair mental health at a population level. Such objective measures, in conjunction with self-reported impacts, offer a comprehensive evidence base which is essential for shaping informed public health policies. Integrating this data with knowledge about infectious diseases equips public health authorities to make informed decisions about how to implement lockdown measures in the most optimal ways possible, that is, balancing the imperative to reduce virus transmission with protecting the mental health of the population from the ramifications of such restrictions. In future pandemics or similar crises, this knowledge may contribute to governments being able to mitigate mental health challenges in the population and provide more targeted and effective mental health care during such exigencies.

A few studies employed an objective measure to assess the impact of the pandemic using the stringency of pandemic policies (i.e., the level of government restriction severity in response to the pandemic) [17–20]. Some studies comprehensively measured the stringency using multiple policy indicators (e.g., school closures, workplace closures, cancellation of public events, restrictions on gatherings, public transportation closures, stay at home requirements, restrictions on internal movement, international travel control) [17, 20] while other studies used levels of lockdown severity only [18, 19]. More stringent policies have been found to be associated with higher levels of psychological distress, lower life evaluation [17], higher levels of depressive symptoms [18], a greater increase in depression symptoms and a lesser symptom reduction from pre-to post-pandemic [19]. Another study found that the stringency of lockdown measures mediated a reduction in the number of self-harm presentations among children and adolescents [20]: the presentations of self-harm among males, children in care, and those who engaged in self-harm due to social isolation were found to be likely to increase with lockdown stringency, whereas presentations among children from deprived neighbourhoods were found to have decreased with more stringent lockdown regulations [20]. However, none of these studies investigated whether varying durations of lockdown impacted mental health differently.

In Australia, lockdowns enforced during the pandemic were strict and lengthy relative to other nations and the duration of the lockdowns differed significantly between the geographical areas of Australia. Between 2020 and 2021, Melbourne experienced the world’s longest lockdown (nearly nine months) and in Sydney, residents were in lockdown for five and half months on average. Although there have been studies examining the impact of the pandemic on mental health among Australian youth, they are limited to cross-sectional studies [14], or they only examined short-term mental health impacts during 2020 [11, 15, 21, 22].

When examining the impact of different pandemic restrictions on the mental health of adolescents, it is crucial to consider potential confounders given that some young people were at higher risk of poor mental health than others during the pandemic. These risk factors included gender [1, 2], lesbian, gay, bisexual, transgender, queer and asexual (LGBTQA) + identity [23], disability diagnoses [24], mental health diagnoses [14, 25], hours of daily screen time and sleep quality [12, 14], social support [2, 26, 27] and social connection [2, 15]. Other established risk factors for poor mental health outcomes, such as socioeconomic disadvantage, racial and cultural discrimination [28, 29], have not been closely examined during the pandemic [2] and also warrant examinations for their confounding effects on the impact of the pandemic restrictions on adolescent mental health.

## Methods

### Study aims

The current study aimed to address the research gaps outlined above by investigating both objectively and subjectively measured impacts of the pandemic restrictions on adolescents’ wellbeing, internalising symptoms, and externalising symptoms in the short- and longer-term. Firstly, we aimed to investigate how the duration of COVID-19 lockdowns implemented in 2020–2021 in the states of New South Wales (NSW: the state in which Sydney is located), and Victoria (VIC: the state in which Melbourne is located), impacted adolescents’ wellbeing, internalising symptoms, and externalising symptoms on two occasions: Time 1: Immediately after lockdown restrictions were lifted in 2021 and schools re-opened; and Time 2: 12 months later in 2022. Secondly, we aimed to investigate adolescents’ subjective experience of the pandemic on their learning, social connection, technology use, and family relationships, and to consider how this affected their wellbeing, internalising symptoms, and externalising symptoms at Time 1 and Time 2. Finally, we aimed to understand the risk and protective factors associated with adolescents’ wellbeing, internalising symptoms, and externalising symptoms during the pandemic by examining the effects of demographic and contextual factors as covariates.

### Study design and participants

Data used in this study comes from a prospective cohort study of the mental health of Australian adolescents, with an embedded randomised-controlled trial of digital prevention intervention for depression (the Future Proofing Study [30]). Participants (*N* = 6,388, mean age = 13.9 years at baseline) complete comprehensive mental health and wellbeing surveys annually for six years. The design, methods and baseline characteristics of the Future Proofing Study are described elsewhere [30].

The current study used a subsample of the Future Proofing Study cohort consisting of NSW and VIC students who completed their baseline surveys in the school term immediately following the 2021 lockdown (October-December 2021). From the total baseline sample of *n* = 6,388, we excluded participants on the basis of date of completion (*n* = 4,881 excluded), location (*n* = 418 excluded), or missing data at Time 1 (*n* = 21 excluded). Participants who resided in areas other than NSW and VIC were excluded to avoid introducing lockdown stringency to models as a potential confounder. Participants from Queensland, South Australia and Western Australia were excluded because they did not experience lockdown measures as stringent as those in NSW and VIC. Participants who completed their baseline survey before October 2021 or after December 2021 were also excluded because the duration and subjective experience of the pandemic lockdowns could differ depending on the timing of data collection. Students who were missing any Time 1 (baseline) data in the final regression model (see model specifications) were also excluded from analyses. All of these exclusion criteria resulted in a final sample of *N* = 1,001. Two years of data were included: Time 1 data were collected in 2021 and Time 2 data were collected 12 months later in 2022. This year-long gap between data collection allowed an exploration of the short- and longer-term impacts of the pandemic restrictions and lockdowns on wellbeing, internalising symptoms, and externalising symptoms. Those participants who provided data about internalising and externalising symptoms at Time 2 (12 months) were included in the relevant follow-up analyses (*n* = 729) with 27.2% lost to follow-up. Similarly, those participants who provided wellbeing outcome data at Time 2 (12 months) were included in the relevant analyses (*n* = 726) with 27.5% lost to follow-up.

### Outcomes

#### Internalising and externalising symptoms

Internalising and externalising symptoms were measured using the Strengths and Difficulties Questionnaire [SDQ; 31]. The SDQ is a 25-item screening questionnaire designed to measure the behavioural, emotional and relationship problems of children and adolescents aged between 4 and 17 years. The questionnaire consists of five subscales, each of which measures hyperactivity, emotional symptoms, conduct problems, peer problems, and prosocial behaviour. Each subscale comprises five items where respondents indicate the extent to which each item applies to them on a 3-point Likert scale, using the options of “Not true”, “Somewhat true” or “Certainly true”. The current study used an internalising symptoms subscale (i.e., the sum of emotional symptoms and peer problems subscales) and externalising symptoms subscale (i.e., the sum of hyperactivity and conduct problems subscales). The total score of each subscale ranges from 0 to 20, and higher scores indicate greater symptoms. The internalising and externalising scales were used because they assess the broader construct of these problems which are likely to be more accurate descriptions than five subscales with low-risk community samples [32]. Both subscales had good internal consistency (Cronbach’s α of 0.82 and 0.83, respectively) [33].

#### Wellbeing

Wellbeing was measured using the short form of the Warwick Edinburgh Mental Wellbeing Scale [SWEMWBS; 34]. Respondents indicate the frequency of seven statements about their feelings and thoughts over the past two weeks using a 5-point Likert scale (“None of the time, “Rarely”, “Some of the time”, “Often” and “All of the time”). The total score is the sum of all seven items, transformed using a conversion table. The total score ranges from 7 to 35, and a higher score indicates a higher level of wellbeing. This scale demonstrated good internal consistency (Cronbach’s α of 0.89) [35].

### Predictors (COVID-19 pandemic measures)

#### Lockdown duration

Information regarding the duration of COVID-19 lockdowns in each NSW Local Government Area (LGA) between 2020 and 2021 was extracted from publicly available COVID-19 related public health orders that were legislated by the NSW Minister for Health and Medical Research under the Public Health Act 2010 [36]. We reviewed all relevant public health orders (e.g., enforced commencement, extension, and cancellation of stay-at-home orders) and then recorded the duration and brief details of each stay-at-home order for all NSW LGAs throughout 2020 and 2021. The total duration of stay-at-home orders (in days) for each LGA was calculated by combining the duration of each order over the two years. There were four different durations of lockdown among the participants i.e., 95 days (10.4%), 130 days (6.3%), 170 days (65.6%) and 262 days (17.7%). The variable was then classified into three categories “less than 150 days”, “150–200 days” and “more than 200 days”. For the entire state of VIC, the total duration of the COVID-19 lockdown between 2020 and 2021 was over 250 days [37].

#### Adolescents’ subjective experience of the pandemic

We measured adolescents’ subjective experience of the pandemic using relevant items from the COVID-19 exposure and impact questionnaire. The data was collected from study participants immediately after the 2021 lockdowns (October–December). The survey items were adapted from an existing questionnaire that assesses perceptions of the pandemic [14, 38] and were developed specifically for the Future Proofing Study. Four items were selected to evaluate participants’ subjective experience of the perceived impact of the pandemic on: (i) learning (“How do you think the pandemic affected your learning?”); (ii) social connection (“How socially connected did you feel towards your friends during the COVID-19 pandemic?”); (iii) technology use (“How much did you use technology e.g. texting, video-chat, to connect with your friends during the pandemic?”); and (iv) relationships with family members (“How has the pandemic affected your relationships with family members at home?”). Respondents indicate the extent to which each item applies to them on a 3-point Likert scale using the options of: “Negatively”, “Not at all” and “Positively” for learning and relationships with family members; “Less connected”, “No change” and “More connected” for social connection; and “Less than usual”, “The same” and “More than usual” for technology use. The participants’ responses were classified into binary categories: “Negative impact” and “Positive impact or no impact”.

### Covariates

Potential confounders in the associations of interest were collected at the Time 1 survey or drawn from existing administrative data. Details of each measure are described with questionnaire items provided in Supplementary Table 1, Additional File 3.

#### Individual characteristics

**Gender identity.** Respondents indicated their current gender identity using the options of “Female”, “Male”, “Non-binary” and “Other”. “Non-binary” and “Other” were combined and categorised into “Gender diverse”.

**LGBTQA + identity.** Participants indicated their sexual orientation using the options of “Heterosexual or straight”, “Gay or lesbian”, “Bisexual”, “Pansexual”, “Asexual”, “Other”, “Not sure” and “Prefer not to say”. “Gay or lesbian”, “Bisexual”, “Pansexual”, “Asexual”, “Other” with valid response were combined and categorised into “Sexuality diverse”. Respondents who indicated that they are either “Gender diverse” (base on their response to the gender identity question) or “Sexuality diverse” were categorised as “LGBTQA + identity”. Other respondents were categorised as “non-LGBTQA + identity”.

**Linguistic diversity.** Respondents used a drop-down list to indicate a language that they speak the most at home. Responses were classified into binary categories: “English” and “Other than English”.

**Country of birth**. Participants indicated their country of birth using a drop-down list. Responses were classified into binary categories: “Born in Australia” and “Born overseas”.

**Perceived family wealth.** Participants indicated perceived level of family wealth by responding to the question ‘How well off you think your family is’?. The response choices are: “Not at all”, “Not very”, Fairly”, “Rather” and “Very” and “Prefer not to say”. “Not at all” and “Not very” were combined into “Low”; “Fairly” was categorised into “Middle”; and “Rather” and “Very” were combined into “High”. “Prefer not to say” category remained as is.

**Mental health diagnoses.** Respondents indicated whether they have ever been diagnosed with any of the following mental health conditions: depression, social phobia, generalised anxiety disorder, obsessive compulsory disorder, panic disorder, separation anxiety disorder, alcohol use disorder, substance use disorder, attention deficit hyperactivity disorder, post-traumatic stress disorder, and schizophrenia/psychosis. Based on the responses, participants were grouped into either: “One or more mental health diagnoses” or “No mental health diagnosis”.

**Disability diagnoses.** Respondents indicated whether they have ever been diagnosed with any of the following conditions: Autism or Asperger’s Syndrome, intellectual disability, specific learning disability, Tourette Syndrome or other chronic tic disorder, cerebral palsy, acquired brain injury, other neurological disability, hearing impairment, and visual impairment. Based on the responses, participants were grouped into either: “One or more disability diagnoses” or “No disability diagnosis”.

#### Social and behavioural characteristics

**Household makeup.** Participants indicated who lives in their home. Responses were categorised into living with: “Two parents”, “Single parent”, “Blended (stepparents)” and “Other”.

**Daily screen time.** Participants indicated their average daily screen time using the option of “0–1 hour”, “1–2 hours”, “2–3 hours”, “3–4 hours”, “4–5 hours”, and “5 + hours”. Responses were classified into binary categories: “Up to 3 hours” and “More than 3 hours”.

**Sleep quality.** Sleep quality was measured using a global score of Pittsburgh Sleep Quality Index where a higher global score indicates poorer sleep quality. This index has demonstrated a high level of internal consistency (Cronbach’s α of 0.83) [PSQI; 39].

**Extroversion.** Extroversion was measured by the extroversion scale of the Big Five Personality Inventory (Cronbach’s α of 0.89) [BFI-10; 40].

**Social support.** Social support was assessed using an abbreviated version of the Schuster Social Support Scale [SSSS; 40]. The SSSS is a 15-item measure of positive and negative interactions with family, friends and spouse. Participants in this study are asked to respond to 10 items chosen from the SSSS to assess interactions with family and friends only. Each item is rated on a 4-point Likert scale ranging from 0 (Never) through to 3 (Often). Scores are interpreted per category, for friends and family, with higher scores on the supportive interaction scales indicative of more supportive interactions, and higher scores on the negative interaction scales indicative of more negative interactions. Positive items had an acceptable level of reliability (Cronbach’s α of 0.64–0.75), while negative items had a lower than acceptable reliability (Cronbach’s α of 0.56–0.74) requiring caution when interpreting outcomes [41].

**School connectedness.** School connectedness was measured using items from the OECD Programme for International Student Assessment [PISA; 42]. Six items are administered, rated on a 4-point Likert scale from 1 (strongly agree) through to 4 (strongly disagree). Total scale scores can range from 6 to 24, with higher scores reflecting greater school connectedness (Cronbach’s α of 0.83) [43].

#### School characteristics

**School sector and location.** Data about school sector (government or non-government) and location (major city or inner regional area) was collected via the My School website [44].

**School socio-economic advantage.** School socio-economic advantage was measured using the Index of Community Socio-Educational Advantage of participants' school [ICSEA; 45].

**Study group allocation.** Because the cohort comes from the Future Proofing Study, a prospective cohort with an embedded randomised-controlled trial of digital prevention intervention for depression, study group allocation (intervention or control) was also adjusted for as a covariate.

#### School-LGA characteristics

**Cultural and linguistic diversity.** The percentage of residents who speak language other than English at participants’ school-LGA, and percentage of residents who were born overseas were each calculated using the recent census data [46].

### Procedure

Participating students and their parents provided opt-in consent to take part in the Future Proofing Study. Students completed online consent and Time 1 and Time 2 surveys during in-class sessions which were facilitated by members of the research team at the Black Dog Institute. Eligible students completed Time 1 surveys immediately after school re-opened between October and December 2021, and Time 2 surveys 12 months later between September and December 2022.

### Statistical analyses

Data preparation, frequencies and descriptive statistics and linear mixed-effects models were performed using SPSS statistical software (version 27). Linear mixed-effects models were performed to test whether the COVID-19 pandemic variables predicted wellbeing, internalising symptoms, and externalising symptoms at Time 1 and Time 2, while adjusting for the potential covariates. A random intercept for school was included in all models described above to account for the cluster sampling used in the current study. The Bonferroni-corrected p value of 0.017 was used as significance threshold in all the models to account for the multiple comparisons (three outcomes). Under the assumption that data were missing random, linear mixed-effects models excluded participants with missing data from analyses relevant to the missing data.

### Model specifications

To first examine the fixed effects of the COVID-19 pandemic variables on the outcomes, five COVID-19 pandemic variables (lockdown duration, subjective experiences of the pandemic on learning, social connection, technology use, and family relationships) were simultaneously entered into the initial models as fixed effects without any potential covariates (model 1). The COVID-19 pandemic variables which were not associated with any of the outcomes based on omnibus tests in model 1 (based on *p* <.017) were excluded from further models. All potential covariates were then added to the models as fixed effects (model 2). To develop more parsimonious models, covariates which were not significantly associated with any of the outcomes based on the omnibus test in model 2 were excluded from further models. All COVID-19 pandemic variables and covariates which were significantly associated with at least one of the outcomes based on the earlier models were simultaneously entered into the final models (model 3). Based on omnibus tests in model 1 (see Supplementary Table 2, Additional File 4), technology use during the pandemic was excluded from further models. Based on omnibus tests in model 2 (see Supplementary Table 3, Additional File 5), the following variables were excluded from the analyses: perceived family wealth, individual-level linguistic diversity and country of birth, a history of mental health diagnosis, household makeup, school sector, study group allocation, and school LGA-level linguistic diversity and country of birth. Participants’ state (NSW or VIC) was also excluded as it was nested within total duration of lockdowns. Finally, significant COVID-19 pandemic variables in model 1 and significant covariates from model 2 were simultaneously entered into the final models as fixed effects to examine their independent associations with the outcomes at Time 1 (model 3). The same final model was then used to model the outcomes at Time 2 for consistency. Using the model, the final Hessian matrix was not positive-definite for internalising symptoms at Time 1, Time 2 or wellbeing at Time 2. Consequently, the models for these three outcomes were re-estimated without the random intercepts, given the possibility of small clustering (this re-analysis resulted in a positive-definite Hessian matrix). As the results of these models remained unchanged irrespective of the inclusion of the random effect, the results with the random effect were reported below.

## Results

### Descriptive statistics

The sample of the current study consisted of 1,001 adolescents (*n* = 824, 82.3% resided in NSW and *n* = 177, 17.7% resided in VIC). Baseline characteristics (individual and contextual factors, internalising symptoms, externalising symptoms, and wellbeing outcomes, and COVID-19 pandemic variables) are summarised in Table 1, Additional File 1.

**Table 1:** Baseline characteristics of the sample (*N* = 1,001)

Characteristics	Statistics
***Individual characteristics***	
Age (years), mean (SD)	14.2 (0.54)
*Gender identity, n (%)*	
Female	453 (45.3)
Male	492 (49.2)
Gender diverse	56 (5.6)
*LGBTQA + identity, n (%)*	
Yes	151 (15.1)
No	850 (84.9)
*Linguistic diversity, n (%)*	
English	921 (92.0)
Other than English	80 (8.0)
*Country of birth, n (%)*	
Born in Australia	912 (91.1)
Born overseas	89 (8.9)
*Perceived family wealth, n (%)*	
Low	69 (6.9)
Middle	316 (31.6)
High	489 (48.9)
Prefer not to say	127 (12.7)
*Mental health diagnoses, n (%)*	
One or more mental health diagnoses	112 (11.2)
No mental health diagnosis	889 (88.8)
*Disability diagnoses, n (%)*	
One or more disability diagnoses	166 (16.6)
No disability diagnosis	835 (83.4)
***Social and behavioural characteristics***	
*Household makeup, n (%)*	
Two parents	816 (81.5)
Single parent	86 (8.6)
Blended (stepparents)	92 (9.2)
Other	7 (0.7)
*Daily screen time, n (%)*	
>3 h	679 (67.8)
≤3 h	322 (32.2)
Sleep quality, mean (SD)	5.9 (3.4)
Extroversion, mean (SD)	6.6 (2.2)
*Social support*	
Supportive interactions with friends, median [IQR]*	3.5 [3.0 to 4.0]
Negative interactions with friends, mean (SD)	1.9 (0.69)
Supportive interactions with family, median [IQR]*	4.0 [3.5 to 4.0]
Negative interactions with family, mean (SD)	2.3 (0.84)
School connectedness, mean (SD)	18.0 (3.5)
***School characteristics***	
*Sector, n (%)*	
Government	497 (49.7)
Non-Government	504 (50.3)
*Location, n (%)*	
Major cities	871 (87.0)
Inner regional	130 (13.0)
ICSEA, mean (SD)	1062 (89)
*Study group allocation, n (%)*	
Intervention	617 (61.6)
Control	384 (38.4)
***School-LGA characteristics***	
Index of Relative Socio-economic Advantage and Disadvantage (IRSAD) (in percentile), mean (SD)	73 (23.7)
Language other than English (in percentage), mean (SD)	26.8 (19.4)
Born overseas (in percentage), mean (SD)	30.5 (15.6)
***Mental health and wellbeing outcomes***	
Internalising symptoms, mean (SD)	5.7 (3.8)
Externalising symptoms, mean (SD)	6.6 (3.8)
Wellbeing, mean (SD)	21.5 (4.7)
***COVID-19 pandemic measures***	
*Total duration of the lockdowns, n (%)*	
<150 days	167 (16.7)
150–200 days	657 (65.6)
>200 days	177 (17.7)
*Perceived impact on learning, n (%)*	
Negative impact	538 (53.7)
Positive or no impact	463 (46.3)
*Perceived impact on social connection, n (%)*	
Negative impact	502 (50.1)
Positive or no impact	499 (49.9)
*Perceived impact on technology use, n (%)*	
Negative impact	88 (8.8)
Positive or no impact	913 (91.2)
*Perceived impact on family relationships, n (%)*	
Negative impact	131 (13.1)
Positive or no impact	870 (86.9)

Note: *Median and inter-quartile range were reported for variables that were not normally distributed

### Primary analyses

Analysis using a linear mixed-effects model was performed with significant COVID-19 pandemic variables from model 1 and significant covariates from model 2 to examine their independent associations with the outcomes at Time 1 and Time 2.

#### COVID-19 pandemic measures

Estimates of fixed effects of all COVID-19 pandemic variables in model 3 on wellbeing, internalising symptoms, and externalising symptoms are summarised in Table 2, Additional File 2. A negative reported subjective experience of the pandemic on learning was significantly associated with externalising symptoms at Time 1 (t = 5.17, df = 980, *p* <.001, d = 0.26), where those who reported that the pandemic negatively affected their learning had greater externalising symptoms compared to those who reported that the pandemic positively affected or did not affect their learning. This association remained at Time 2 (t = 2.72, df = 708, *p* =.007, d = 0.19). A negative subjective experience of the pandemic on social connection was significantly associated with internalising symptoms at Time 2 (t = 3.20, df = 709, *p* =.001, d = 0.19), but not at Time 1 (t = 2.32, df = 981, *p* =.20), where those who reported feeling less socially connected towards friends than usual during the pandemic had greater internalising symptoms 12 months after lockdowns lifted, compared with those who reported feeling more socially connected or had no change in social connection. A negative subjective experience of the pandemic on family relationships was not significantly associated with any of the outcomes at Time 1 (Internalising: t = 1.95, df = 981, *p* =.052; Externalising: t = 0.40, df = 975, *p* =.689; Wellbeing: t = 0.36, df = 974, *p* =.722) or Time 2 (Internalising: t = 0.82, df = 709, *p* =.411; Externalising: F = 0.84, df = 705, *p* =.401; Wellbeing: t = 0.08, df = 706, *p* =.939). Similarly, lockdown duration was not associated with any of the outcomes at Time 1 (Internalising: F = 0.071, df = 981, *p* =.932; Externalising: F = 0.757, df = 51, *p* =.474; Wellbeing: F = 1.172, df = 43, *p* =.319), or Time 2 (Internalising: F = 0.228, df = 709, *p* =.796; Externalising: F = 0.740, df = 23, *p* =.488; Wellbeing: F = 0.405, df = 706, *p* =.667).

**Table 2:** Results of fixed effects of COVID-19 pandemic variables and covariates for outcomes variables

	Internalising symptoms						Externalising symptoms						Wellbeing					
		Time 1			Time 2			Time 1			Time 2			Time 1			Time 2	
	Est.	Test (df)	*P*	Est.	Test (df)	*p*	Est.	Test (df)	*p*	Est.	Test (df)	*p*	Est.	Test (df)	*p*	Est.	Test (df)	*p*
**Perceived impact** (Neg vs. Pos/No)																		
**Learning**	0.292	t = 1.83 (981)	*p* =.067	-0.006	t=-0.02 (709)	*p* =.980	0.993	t = 5.17 (980)	*p* <.001↑ **(d = 0.26)**	0.701	t = 2.72 (708)	*p* =.007↑ **(d = 0.19)**	-0.451	t=-1.96 (980)	*p* <.050	-0.583	t=-1.66 (706)	*p* =.097
**Social connection**	0.362	t = 2.32 (981)	*p* =.020	0.728	t = 3.20 (709)	p =.001↑ **(d = 0.19)**	-0.116	t = 0.62 (981)	*p* =.538	0.403	t = 1.60 (709)	*p* =.111	-0.387	t=-1.72 (981)	*p* =.085	-0.152	t=-0.44 (706)	*p* =.660
Family relationships	0.485	t = 1.95 (981)	*p* =.052	0.309	t = 0.82 (709)	*p* =.411	0.12	t = 0.40 (975)	*p* =.689	0.349	t = 0.84 (705)	*p* =.401	0.127	t = 0.36 (974)	*p* =.722	-0.044	t=-0.08 (706)	*p* =.939
**Lockdown duration** (vs. < 150 days)																		
> 200 days	-0.119	t=-0.34 (981)	*p* =.731	0.362	t = 0.68 (709)	*p* =.499	-0.565	t=-1.23 (50)	*p* =.224	-0.852	t=-1.22 (20)	*p* =.238	0.837	t = 1.53 (43)	*p* =.133	0.704	t = 0.87 (706)	*p* =.386
150–200 days	-0.053	t=-0.17 (981)	*p* =.863	0.227	t = 0.46 (709)	*p* =.645	-0.376	t=-0.93 (49)	*p* =.358	-0.554	t=-0.86 (19)	*p* =.402	0.577	t = 1.20 (42)	*p* =.238	0.569	t = 0.76 (706)	*p* =.447
**Gender identity** (vs. Male)																		
Female	1.751	t = 10.60 (981)	*p* <.001↑ **(d = 0.46)**	1.837	t = 7.53 (709)	*p* <.001↑ **(d = 0.48)**	0.149	t = 0.72 (248)	*p* =.473	0.221	t = 0.76 (148)	*p* =.446	-0.905	t=-3.65 (216)	*p* <.001↓ **(d=-0.19)**	-1.061	t=-2.88 (706)	*p* =.004↓ **(d=-0.21)**
Gender diverse	0.939	t = 2.41 (981)	*p* =.016↑ **(d = 0.25)**	0.402	t = 0.72 (709)	*p* =.471	0.136	t = 0.29 (978)	*p* =.772	-1.061	t=-1.72 (708)	*p* =.087	-1.199	t=-2.13 (977)	*p* =.033	-0.415	t=-0.49 (706)	*p* =.621
**LGBTQA + identity** Yes (vs. No)	0.822	t = 3.35 (981)	*p* <.001↑ **(d = 0.22)**	1.336	t = 3.69 (709)	*p* <.001↑ **(d = 0.35)**	0.99	t = 3.35 (979)	*p* <.001↑ **(d = 0.26)**	1.106	t = 2.76 (708)	*p* =.006↑ **(d = 0.30)**	-0.098	t=-0.28 (978)	*p* =.782	-0.883	t=-1.62 (706)	*p* =.106
**Disability diagnosis** One or more (vs. No)	0.302	t = 1.41 (981)	*p* =.158	0.703	t = 2.25 (709)	*p* =.025	1.133	t = 4.39 (980)	*p* <.001↑ **(d = 0.29)**	1.257	t = 3.64 (708)	*p* <.001↑ **(d = 0.34)**	-0.241	t=-0.78 (979)	*p* =.434	-1.137	t=-2.42 (706)	*p* =.016↓ **(d=-0.22)**
**Extroversion**	-0.239	t=-5.87 (981)	*p* <.001↓ **(d=-0.06)**	-0.276	t=-4.61 (709)	*p* <.001↓ **(d=-0.07)**	0.256	t = 5.20 (980)	*p* <.001↑ **(d = 0.07)**	0.122	t = 1.83 (708)	*p* =.067	0.198	t = 3.37 (980)	*p* <.001↑ **(d = 0.04)**	0.257	t = 2.85 (706)	*p* =.004↑ **(d = 0.05)**
**Daily screen time** > 3 h (vs. ≤ 3 h)	0.298	t = 1.78 (981)	*p* =.075	0.29	t = 1.20 (709)	*p* =.232	1.034	t = 5.11 (981)	*P* <.001↑ **(d = 0.27)**	0.621	t = 2.31 (709)	*p* =.021	-0.95	t=-3.93 (981)	*p* <.001↓ **(d=-0.20)**	-0.304	t=-0.83 (706)	*p* =.407
**Sleep quality**	0.273	t = 10.08 (981)	*p* <.001↑ **(d = 0.07)**	0.131	t = 3.30 (709)	*p* =.001↑ **(d = 0.03)**	0.343	t = 10.51 (981)	*P* <.001↑ **(d = 0.09)**	0.198	t = 4.48 (709)	*p* <.001↑ **(d = 0.05)**	-0.254	t=-6.50 (981)	*p* <.001↓ **(d=-0.05)**	-0.125	t=-2.08 (706)	*p* =.037
**Supportive interactions with friends**	-0.400	t=-3.19 (981)	*p* =.001↓ **(d=-0.11)**	-0.006	t=-0.03 (709)	*p* =.976	-0.384	t=-2.54 (981)	*P* =.011↓ **(d=-0.10)**	-0.490	t=-2.39 (709)	*p* <.017↓ **(d=-0.13)**	1.324	t = 7.33 (981)	*p* <.001↑ **(d = 0.28)**	0.454	t = 1.62 (706)	*p* =.105
**Negative interactions with friends**	0.103	t = 0.82 (981)	*p* =.411	-0.278	t=-1.46 (709)	*p* =.144	0.441	t = 2.92 (976)	*p* =.004↑ **(d = 0.11)**	-0.157	t=-0.75 (704)	*p* =.456	0.214	t = 1.19 (975)	*p* =.236	-0.22	t=-0.77 (706)	*p* =.443
**Supportive interactions with family**	0.195	t = 1.37 (981)	*p* =.170	0.241	t = 1.12 (709)	*p* =.262	-0.464	t=-2.71 (980)	*p* =.007↓ **(d=-0.12)**	-0.232	t=-0.97 (709)	*p* =.332	0.832	t = 4.06 (980)	*p* <.001↑ **(d = 0.18)**	0.622	t = 1.89 (706)	*p* =.059
**Negative interactions with family**	0.31	t = 2.68 (981)	*p* =.008↑ **(d = 0.08)**	0.46	t = 2.64 (709)	*p* =.009↑ **(d = 0.12)**	0.751	t = 5.39 (975)	*p* <.001↑ **(d = 0.20)**	0.662	t = 3.44 (703)	*p* <.001↑ **(d = 0.18)**	-0.405	t=-2.43 (974)	*p* =.015↓ **(d=-0.09)**	-0.304	t=-1.15 (706)	*p* =.250
**School** **connectedness**	-0.376	t=-12.69 (981)	*p* <.001↓ **(d=-0.10)**	-0.297	t=-6.48 (709)	*p* <.001↓ **(d=-0.08)**	-0.102	t=-2.86 (977)	*p* =.004↓ **(d=-0.03)**	-0.091	t=-1.81 (705)	*p* =.071	0.332	t = 7.78 (976)	*p* <.001↑ **(d = 0.07)**	0.27	t = 3.92 (706)	*p* <.001↑ **(d = 0.05)**
**School location** Inner regional (vs. Major cities)	0.199	t = 0.64 (981)	*p* =.521	-0.374	t=--0.77 (709)	*p* =.443	0.331	t = 0.78 (34)	*p* =.439	-0.247	t=-0.38 (17)	*p* =.708	-1.428	t=-2.84 (29)	*p* =.008↓ **(d=-0.31)**	-0.284	t=-0.39 (706)	*p* =.700
**ICSEA**	-0.001	t=-0.86 (981)	*p* =.391	-0.004	t=-2.27 (709)	*p* =.023	-0.001	t=-0.83 (36)	*p* =.409	-0.001	t=-0.65 (17)	*p* =.521	-0.005	t=-2.71 (31)	*p* =.011↓ **(d=-0.01)**	0	t = 0.05 (706)	*p* =.964

Significant p values (*p* <.017) are in bold and italics, indicated with ↑ for a positive relationship or ↓ for a negative relationship, and provided with an effect size calculated by Cohen d; Time 1 refers to between October and December 2021 when pandemic lockdowns were lifted and school re-opened; Time 2 refers to approximately 12 months later between September and December 2022

#### Risk and protective factors—covariates

Estimates of the fixed effects of all individual, social, behavioural and school-level factors in model 3 on wellbeing, internalising symptoms, and externalising symptoms are summarised in Table 2, Additional File 2. Risk factors identified for higher internalising symptoms at both Time 1 and Time 2 were female gender, LGBTQA + identity, poor sleep quality, and greater negative interactions with family. Gender diverse identity was associated with higher internalising symptoms at Time 1 only. Protective factors associated with lower internalising symptoms at both Time 1 and Time 2 were higher extroversion and higher school connectedness. Higher supportive interactions with friends was associated with lower internalising symptoms at Time 1 only.

Risk factors identified for higher externalising symptoms at both Time 1 and Time 2 were LGBTQA + identity, reported disability diagnosis, poor sleep quality, and greater negative interactions with family. Higher extroversion, more than 3 hours of daily screen time, and greater negative interactions with friends were associated with higher externalising symptoms at Time 1 only. A protective factor associated with lower externalising symptoms at both Time 1 and Time 2 was higher positive interactions with friends. Higher positive interactions with family and higher school connectedness were associated with lower externalising symptoms at Time 1 only.

A risk factor identified for lower wellbeing at both Time 1 and Time 2 was female gender. More than 3 hours of daily screen time, poor sleep quality, greater negative interactions with family, residing in an inner regional area, and higher school ICSEA were associated with lower wellbeing at Time 1 only. A reported disability diagnosis was associated with lower wellbeing at Time 2 only. Protective factors identified for wellbeing at both Time 1 and Time 2 were higher extroversion and higher school connectedness. Higher positive interactions with friends and family were associated with higher wellbeing at Time 1 only.

## Discussion

Our main findings indicate that adolescents’ negative subjective experience of the pandemic, specifically in terms of social connection and learning, was associated with higher internalising and externalising symptoms following lockdowns. However, the amount of time that adolescents spent in lockdown and their subjective experience of the pandemic on their family relationships were not associated with wellbeing, internalising symptoms, or externalising symptoms.

Contrary to public concerns about the toll of prolonged lockdowns on mental health [1, 2, 47, 48], and despite large differences in lockdown durations between participants (< 150 days, 150–200 days and > 200 days), longer lockdowns were not associated with a deterioration in wellbeing, nor with an increase in internalising or externalising symptoms among Australian adolescents immediately after the lockdowns lifted or 12 months later. These findings highlight the importance of young people’s subjective experiences of pandemic restrictions in relation to their mental health. The importance of young people’s diverse experiences during the pandemic and the relationship of these experiences with mental health has been highlighted in the literature [2, 49]. For example, for many young people, school closure meant that they missed face-to-face social interactions and experienced increased loneliness, while for others, it meant being away from various stressful aspects of in-person schooling such as negative peer interactions, bullying, and disruptive classroom environments [49]. Although the current study measured adolescents’ wellbeing, internalising symptoms, and externalising symptoms when schools reopened rather than during school closures, the impacts of these diverse experiences during the lockdowns may have had carry-on effects for adolescents’ mental health following the return to school.

Our findings highlight some of the aspects of young people’s lives that were important for their mental health during the pandemic, i.e., learning and social connection. Consistent with previous research [15, 26, 50], we found that adolescents who reported a negative impact of the pandemic on their learning had significantly higher externalising symptoms than those who did not, both when school reopened and 12 months later. One of the main changes associated with the lockdown for school students was the transition to online learning. Difficulty with online learning was one of the most commonly reported challenges faced by young people during the pandemic [2]. To successfully learn online, students needed not only material resources such as reliable internet, computer and distraction-free home environment, but also social resources such as parents and teachers who were available to provide guidance and support for online learning [51]. Disparities in students’ access to these resources were observed during the pandemic between Australian schools based on their level of socio-educational advantage [52]. These disparities included teachers’ inequitable access to professional training to support their students’ online learning [52]. In addition to these necessary resources, students were required to self-direct their learning to compensate for the lack of instructor support [51]. The greater demands for self-directed learning might have been particularly challenging for students with externalising problems. A US study estimated the pandemic digital divide could result in the loss of 7–14 months’ worth learning and prospective annual earnings of $110 billion, as well as the dropouts of 232,000 high school students [51]. In possible future emergencies with protracted lockdowns, public health resources need to be allocated to address this unequitable access to vital resources among school students to support their learning, mental health, and future.

Interestingly, we found that adolescents who reported that the pandemic had a negative impact on their social connections had significantly greater internalising symptoms 12 months after lockdowns were lifted (Time 2), but this association was not significant at school re-opening (Time 1). One possible explanation is that the effect of negative social connection on young people’s internalising symptoms might have been latent and thus not apparent when school re-opened. However, this negative impact might have become evident and observable following exposure to more social interactions in the subsequent year when school re-opened, a phenomenon which Wade et al. [53] describes as a *sleeper effect*. In a qualitative study [49], young people reported that their anxiety and stress was associated with adjusting to being back in the school environment, which was related, in part, to difficult peer interactions. Hence, in future situations when young people are restricted from interacting in person, mental health support and interventions could focus on assisting them to build and maintain social connections, as well as to develop social and interpersonal skills.

In sum, our findings suggest that lengthier lockdowns did not make statistically significant differences in the longer-term mental health and wellbeing of adolescents, but that the negative impact of lockdowns on their mental health can be minimised by supporting their transition into unfamiliar learning environments and by equipping them with the skills and tools required to connect with their social networks. Given that catastrophic natural disasters are becoming more common due to the escalating effects of climate change, lockdowns may well be required more often in the future. Therefore, it is vital to integrate these targeted strategies for mental health into future public health responses. In addition, considering the possibility of sleeper effects of the pandemic on young people’s mental health post-lockdown, policy makers also need to focus on providing post-lockdown support, equipping schools to intensify their efforts to assist students to rebuild their social connections and catch up with the learning that was interrupted by the pandemic. The main protective factors that we identified were school connectedness and positive interactions with friends and family, further emphasising the importance of social skills and peer connectivity as a buffer against the development of mental health symptoms associated with the pandemic.

Our results also demonstrated that various established risk factors for poorer mental health continued to be associated with lower wellbeing and higher internalising and externalising symptoms during and following the pandemic. In our sample, we found that females were at a higher risk of internalising symptoms during and following the pandemic, relative to males. This is not surprising, as studies have shown that females are more susceptible to experiencing symptoms of depression due to social stressors [54], and that they tend to place higher importance on peer relationships and support compared to males [55].

The other demographic group at higher risk of mental health symptoms were adolescents who reported LGBTQA + identity. Higher internalising and externalising symptoms among LGBTQA + young people during and following the pandemic could have been due to lockdown isolation from in-person identity-specific social connections and support, as well as confinement at home with potentially unsupportive families [23].

We also found that adolescents who reported one or more disability diagnoses had higher externalising symptoms at both time points, and lower wellbeing at Time 2. The reported higher externalising symptoms during the lockdown may be because many Australian students with a disability usually access counsellors and educational support at school which they cannot easily obtain at home [26]. During school closures, these students may have lost access to this tailored support, structured environment [56], and relationship and engagement with their teachers, which may have been important for their learning [52]. The higher externalising symptoms reported by adolescents with disabilities might therefore have been associated with the loss of these resources provided at school. The provision of specialised resources for their family to support their children’s home learning would have helped achieve the continuity of support for these students. However, many parents were not provided with such support and resources during the pandemic [24, 56]. The higher externalising symptoms and lower wellbeing reported following the pandemic might have been related to a degree of ‘back to school stress’ for these young people. One study conducted in the UK found evidence that wellbeing among adolescents with disability improved during the lockdowns compared to pre-pandemic levels [57]. It is also important to note that neurological and developmental disorders are often comorbid with externalising symptoms [58]. Considering the comorbidity, adolescents with disability might have been already at a risk of externalising symptoms prior to the pandemic. School closures and remote learning resulting from the pandemic may well have exacerbated their existing risk.

Together, our findings highlight the importance of providing tailored support for these higher risk groups of adolescents. In future situations, public health resources need to be allocated to schools to enable staff to provide additional support to these vulnerable groups and their families.

Further, we found that poorer sleep quality was a risk factor associated with higher internalising and externalising symptoms both upon school reopening and 12 months later, as well as lower level of wellbeing upon returning to school. Adolescents who engaged in more than 3 hours of daily screen time were also found to have higher externalising symptoms and lower wellbeing at school re-opening. Our findings align with previous research demonstrating an association between adolescent mental health, and sleep quality and screen use during the pandemic [12, 14]. These factors are potentially modifiable and are therefore viable targets for interventions.

### Strengths and limitations

There are many strengths in this study. First, mental health and wellbeing outcomes were measured using validated instruments which provide a reliable proxy for mental health conditions. Although data from diagnostic interviews would have been more rigorous, this was not feasible given the scale of the cohort. Second, our data was collected at a later stage of the pandemic– that is, immediately after the end of the 2021 lockdown, and post-lockdown in 2022– whereas currently available evidence on the impact of the pandemic on mental health was largely collected at an early stage of the pandemic (i.e., 2020). Consequently, our study provides a more comprehensive picture of the short- and longer-term impacts of the pandemic on adolescent mental health. Finally, this study included longitudinal data hence providing an insight into how aspects of the pandemic relate to changes in adolescent mental health over a 12-month period.

The current study also has some limitations. As the baseline data were collected after the lockdowns in 2021, we were unable to examine the changes in mental health and wellbeing outcomes from pre-pandemic to during the pandemic. The timing of the data collection might also have increased the risk of recall bias when reporting subjective experiences, because participants were asked to retrospectively report the experiences they had from during the lockdowns when they had returned to school at the end of 2021. Also, the duration between the end of lockdown and the completion of the Time 1 surveys varied by approximately two months among participants. The variance might have influenced how accurately they reported their subjective experiences of the lockdowns. Further, the different lockdown durations mandated in Australia were not randomly allocated, and may have been associated with factors that could not be fully accounted for in the analyses. Moreover, the majority of our sample resided in major cities of NSW. It resulted in the < 150 days and > 200 days groups having smaller number of participants than 150–200 days group. The uneven distribution of participants in lockdown duration groups and the lack of a no-lockdown group might have influenced the results. In addition, most of the significant associations reported here are small in effect size. Although estimated differences in mental health and wellbeing outcomes between groups are statistically significant, they do not necessarily infer clinically significant differences. In addition, cross-sectional findings (at school re-opening) are limited to associations at a single point in time, and changes in associations cannot be examined. Moreover, attrition rate was about 25% for the analyses of the 12-month data and may have been non-random. Finally, there is a possibility of bidirectionality between identified pandemic-related risk factors and mental health outcomes (i.e., subjective impact on learning and externalising symptoms, and subjective impact on social connection and internalising symptoms). This bidirectionality was not examined in the current study.

## Conclusions

To our knowledge, this is the first study to examine the association between lockdown duration and wellbeing, internalising symptoms, and externalising symptoms in adolescents. We did not find evidence to suggest that the total time spent in lockdown was associated with higher internalising or externalising symptoms, or with lower wellbeing. However, we did find evidence to suggest that adolescents’ negative subjective experiences of the lockdowns on their social connections and learning was associated with higher internalising and externalising symptoms, respectively. To protect young people’s mental health from the longer-term impacts of lockdowns in future similar scenarios, it is critical for policy makers to provide schools and families with the support to assist young people to adapt to new routines and learning environments, and to build and maintain social connections and social and interpersonal skills. This study also identified some groups of adolescents at elevated risk of higher internalising and externalising symptoms and lower wellbeing suggesting that tailored support should be provided to female youth, LGBTQA + youth, and adolescents with disabilities in future situations similar to the COVID-19 pandemic. Interventions and programs to support adolescent mental health could also focus on addressing the risk factors identified in this study such as poor sleep quality and extended screen use, as well as on strengthening protective factors such as family and peer support and a sense of belonging in schools. As the effects of climate change and international insecurity continue to present challenges, especially for young people, our findings play a vital role in arguing for better integration of mental health care when preparing for future pandemics and other similar public health crises.

### Electronic supplementary material

Below is the link to the electronic supplementary material.

Supplementary Material 1

Supplementary Material 2

Supplementary Material 3

## Data Availability

The datasets used and analysed during the current study are not publicly available because we do not have approval from the ethics committees to do so, and we do not wish to compromise individual privacy. No datasets were generated or analysed during the current study.

## Abbreviations

NSW: New South Wales
VIC: Victoria
LGA: Local Government Area
ICSEA: The Index of Community Socio-Education Advantage
LGBTQA: Lesbian, Gay, Bisexual, Transgender, Queer and Asexual
