# Supplementary material for: What aspects of the pandemic had the greatest impact on adolescent mental health: duration of lockdown or subjective experience?
Source: Child Adolesc Psychiatry Ment Health. 2024 Jun 1;18:63. doi: 10.1186/s13034-024-00759-3 (PMC11144333; doi:10.1186/s13034-024-00759-3)
Supplement: Supplementary file 1 — Supplementary Material 1 [file 13034_2024_759_MOESM1_ESM.docx]

**Supplementary Table 1** Online self-report survey items

| **Measures** | **ID** | **Questions/scoring** |
| --- | --- | --- |
| Strength and Difficulties Questionnaire (SDQ) | SDQ1 | I try to be nice to other people. I care about their feelings. |
|  | SDQ2 | I am restless, I cannot stay still for long. |
|  | SDQ3 | I get a lot of headaches, stomach-aches or sickness. |
|  | SDQ4 | I usually share with others, for example games or food. |
|  | SDQ5 | I get very angry and often lose my temper. |
|  | SDQ6 | I would rather be alone than with people of my age. |
|  | SDQ7 | I usually do as I am told. |
|  | SDQ8 | I worry a lot. |
|  | SDQ9 | I am helpful if someone is hurt, upset or feeling ill. |
|  | SDQ10 | I am constantly fidgeting or squirming. |
|  | SDQ11 | I have one good friend or more. |
|  | SDQ12 | I fight a lot. I can make other people do what I want. |
|  | SDQ13 | I am often unhappy, depressed or tearful. |
|  | SDQ14 | Other people my age generally like me. |
|  | SDQ15 | I am easily distracted, I find it difficult to concentrate. |
|  | SDQ16 | I am nervous in new situations. I easily lose confidence. |
|  | SDQ17 | I am kind to younger children. |
|  | SDQ18 | I am often accused of lying or cheating. |
|  | SDQ19 | Other children or young people pick on me or bully me. |
|  | SDQ20 | I often volunteer to help others (parents, teachers, children). |
|  | SDQ21 | I think before I do things. |
|  | SDQ22 | I take things that are not mine from home, school or elsewhere. |
|  | SDQ23 | I get along better with adults than with people my own age. |
|  | SDQ24 | I have many fears, I am easily scared. |
|  | SDQ25 | I finish the work I'm doing. My attention is good. |
|  | Emotional Problems | Aggregate score: based on items 'SDQ3', 'SDQ8', 'SDQ13', 'SDQ16', 'SDQ24' |
|  | Conduct Problems | Aggregate score: based on items 'SDQ5', 'SDQ7', 'SDQ12', 'SDQ18', 'SDQ22' |
|  | Hyperactivity | Aggregate score: based on items 'SDQ2', 'SDQ10', 'SDQ15', 'SDQ21', 'SDQ25' |
|  | Peer Problems | Aggregate score: based on items 'SDQ6', 'SDQ11', 'SDQ14', 'SDQ19', 'SDQ23' |
|  | Prosocial | Aggregate score: based on items 'SDQ1', 'SDQ4', 'SDQ9', 'SDQ17', 'SDQ20' |
|  | Total Difficulties Score | Summing scores (subscales 1-4), except the prosocial scale. |
|  | Externalising subscale | Aggregate score: sum of Conduct Problems and Hyperactivity subscales |
|  | Internalising subscale | Aggregate score: sum of Emotional Problems and Peer Problems subscales |
| Short-form Warwick-Edinburgh Mental Well-being Scale (SWEMWBS) | SWEMWBS1 | I've been feeling optimistic about the future. |
|  | SWEMWBS2 | I've been feeling useful. |
|  | SWEMWBS3 | I've been feeling relaxed. |
|  | SWEMWBS4 | I've been dealing with problems well. |
|  | SWEMWBS5 | I've been thinking clearly. |
|  | SWEMWBS6 | I've been feeling close to other people. |
|  | SWEMWBS7 | I've been able to make up my own mind about things. |
|  | Well-being Total Score | Aggregate score: based on sum of items 'SWEMWBS1' to 'SWEMWBS7' |
|  | Well-being Metric Score | Metric conversion of total score. |
| COVID-19 experience and exposure questionnaires | COVID16 | How do you think the pandemic affected your learning? |
|  | COVID18 | How socially connected did you feel toward your friends during the COVID-19 pandemic? |
|  | COVID20 | How much did you use technology (e.g. texting, video-chat) to connect with your friends during the pandemic? |
|  | COVID21 | How has the pandemic affected your relationships with family members at home? |
| Big Five Inventory-10  (BFI-10) | BFI1 | ...is reserved |
|  | BFI2 | ...is generally trusting |
|  | BFI3 | ...tends to be lazy |
|  | BFI4 | ...is relaxed, handles stress well |
|  | BFI5 | ...has few artistic interests |
|  | BFI6 | ...is outgoing, sociable |
|  | BFI7 | ...tends to find fault with others |
|  | BFI8 | ...does a thorough job |
|  | BFI9 | ...gets nervous easily |
|  | BFI10 | ...has an active imagination |
|  | Extroversion | Aggregate score: based on items 'BFI1', 'BFI6' |
| Pittsburgh Sleep Quality Index (PSQI) | PSQI1 | When have you usually gone to bed at night? Please select AM/PM in your answer. |
|  | PSQI2 | How long has it usually taken you to fall asleep each night? |
|  | PSQI3 | When have you usually gotten out of bed in the morning? |
|  | PSQI4 | In total, how many hours of actual sleep have you usually had each night? (This might be less than the number of hours you usually spent in bed.) |
|  | For all PSQI5 questions | During the past month, how often have you had trouble sleeping because you |
|  | PSQI5A | Cannot get to sleep within 30 minutes |
|  | PSQI5B | Wake up in the middle of the night or early morning |
|  | PSQI5C | Have to get up to use the bathroom |
|  | PSQI5D | Cannot breathe comfortably |
|  | PSQI5E | Cough or snore loudly |
|  | PSQI5F | Feel too cold |
|  | PSQI5G | Feel too hot |
|  | PSQI5H | Have bad dreams |
|  | PSQI5I | Have pain |
|  | PSQI5J | Other reason(s) |
|  | PSQI5JR | Please describe the reason(s). |
|  | PSQI6 | How would you rate your sleep quality overall? |
|  | PSQI7 | How often have you taken medicine to help you sleep? |
|  | PSQI8 | How often have you had trouble staying awake during class, eating meals, or engaging in social activity? |
|  | PSQI9 | How much of a problem has it been for you to keep up enthusiasm to get things done? |
|  | Subjective Sleep Quality | Aggregate score: based on item 'PSQI6'. |
|  | Sleep Latency | Aggregate score: based on items 'PSQI2', 'PSQI5A'. |
|  | Sleep Duration | Aggregate score: based on item 'PSQI4'. |
|  | Habitual Sleep Efficiency | Aggregate score: based on items 'PSQI1', 'PSQI3', 'PSQI4'. |
|  | Sleep Disturbances | Aggregate score: based on items 'PSQI5B', 'PSQI5C', 'PSQI5D', 'PSQI5E', 'PSQI5F', 'PSQI5G', 'PSQI5H', 'PSQI5I', 'PSQI5J'. |
|  | Sleep Medication | Aggregate score: based on item 'PSQI7'. |
|  | Daytime Dysfunction | Aggregate score: based on items 'PSQI8', reversed 'PSQI9’. |
|  | Global PSQI Score | Aggregate score: based on all the PSQI aggregate scores. |
| Schuster Social Support Scale (SSSS) | SSSS1 | How often do friends make you feel cared for? |
|  | SSSS2 | How often do friends express interest in how you are doing? |
|  | SSSS3 | How often do friends make too many demands on you? |
|  | SSSS4 | How often do friends criticise you? |
|  | SSSS5 | How often do friends create tensions or arguments with you? |
|  | SSSS6 | How often do family make you feel cared for? |
|  | SSSS7 | How often do family express interest in how you are doing? |
|  | SSSS8 | How often do family make too many demands on you? |
|  | SSSS9 | How often do family criticise you? |
|  | SSSS10 | How often do family create tensions or arguments with you? |
|  | Positive interactions with friends | Aggregate score: based on items ‘SSSS1’ and ‘SSSS2’. |
|  | Negative interactions with friends | Aggregate score: based on items ‘SSSS3’, ‘SSSS4’, ‘SSSS5’. |
|  | Positive interactions with family | Aggregate score: based on items ‘SSSS6’, ‘SSSS7’. |
|  | Negative interactions with family | Aggregate score: based on items ‘SSSS8’, ‘SSSS9’, ‘SSSS10’. |
| Items from OECD Programme for International Student Assessment (PISA) | SCHC1 | I make friends easily at school. |
|  | SCHC2 | I feel like I belong at school. |
|  | SCHC3 | Other students seem to like me. |
|  | SCHC4 | I feel like an outsider (or left out of things) at school. |
|  | SCHC5 | I feel awkward and out of place in my school. |
|  | SCHC6 | I feel lonely at school. |
|  | Total score | Aggregated score: based on items reversed ‘SCHC1’, reversed ‘SCHC2’, reversed ‘SCHC3’, ‘SCHC4’, ‘SCHC5’. |
