# Supplementary material for: What aspects of the pandemic had the greatest impact on adolescent mental health: duration of lockdown or subjective experience?
Source: Child Adolesc Psychiatry Ment Health. 2024 Jun 1;18:63. doi: 10.1186/s13034-024-00759-3 (PMC11144333; doi:10.1186/s13034-024-00759-3)
Supplement: Supplementary file 2 — Supplementary Material 2 [file 13034_2024_759_MOESM2_ESM.docx]

**Supplementary Table 2** Omnibus test results of the pandemic measures on outcome variables at Time 1

|  | **Internalising symptoms** | |  | **Externalising symptoms** | |  | **Wellbeing** | |
| --- | --- | --- | --- | --- | --- | --- | --- | --- |
|  | **Test (df)** | **p** |  | **Test (df)** | **p** |  | **Test (df)** | **p** |
| Perceived impact on Learning | F=26.2 (1,996) | p<0.001* |  | F=66.3 (1,1000) | p<0.001* |  | F=25.0 (1,998) | p<0.001* |
| Perceived impact on Social connection | F=20.7 (1,1002) | p<0.001* |  | F=0.2 (1,1004) | p=0.671 |  | F=12.7 (1,1002) | p<0.001* |
| Perceived impact on Technology use | F=3.2 (1,996) | p=0.073 |  | F=4.0 (1,998) | p=0.045 |  | F=0.1 (1,997) | p=0.731 |
| Perceived impact on Family relationships | F=35.7 (1,992) | p<0.001* |  | F=51.4 (1,994) | p<0.001* |  | F=20.8 (1,993) | p<0.001* |
| Lockdown duration | F=4.2 (2,128) | p=0.017 |  | F=5.7 (2,82) | p=0.005* |  | F=5.0 (2,118) | P<0.008* |

*Note:* Significant p values (p<0.017) are in indicated with *
