# Supplementary material for: What aspects of the pandemic had the greatest impact on adolescent mental health: duration of lockdown or subjective experience?
Source: Child Adolesc Psychiatry Ment Health. 2024 Jun 1;18:63. doi: 10.1186/s13034-024-00759-3 (PMC11144333; doi:10.1186/s13034-024-00759-3)
Supplement: Supplementary file 3 — Supplementary Material 3 [file 13034_2024_759_MOESM3_ESM.docx]

**Supplementary Table 3** Omnibus test results of the pandemic measures and covariates on outcome variables at Time 1

|  | **Internalising symptoms** | |  | **Externalising symptoms** | |  | **Wellbeing** | |
| --- | --- | --- | --- | --- | --- | --- | --- | --- |
|  | **Test (df)** | **P** |  | **Test (df)** | **p** |  | **Test (df)** | **p** |
| Perceived impact on learning | F=3.4 (1,968) | p=0.066 |  | F=26.1 (1,968) | p<0.001* |  | F=3.7 (1,968) | p=0.056 |
| Perceived impact on  social connection | F=5.0 (1,968) | p=0.026 |  | F=0.3 (1,968) | p=0.555 |  | F=3.1 (1,968) | p=0.078 |
| Perceived impact on  family relationships | F=3.8 (1,965) | p=0.053 |  | F=0.4 (1,963) | p=0.522 |  | F=0.3 (1,968) | p=0.571 |
| Lockdown duration | F=0.4 (2,21) | p=0.673 |  | F=1.1 (2,27) | p=0.360 |  | F=1.4 (2,968) | p=0.239 |
| Gender identity | F=47.9 (2,675) | <0.001* |  | F=0 (2, 705) | p=0.964 |  | F=4.2 (2, 968) | p=0.015* |
| LGBTQA+ identity | F=10.0 (1,968) | p=0.002* |  | F=10.3 (1, 967) | p=0.001* |  | F=0.1 (1, 968) | p=0.778 |
| Perceived family wealth | F=1.1 (3, 961) | p=0.354 |  | F=0.5 (3, 962) | p=0.648 |  | F=2.9 (3, 968) | p=0.033 |
| Linguistic diversity | F=0.1 (1, 933) | p=0.713 |  | F=0 (1, 933) | p=0.844 |  | F=0.1 (1, 968) | p=0.699 |
| Country of birth | F=0 (1,967) | p=0.992 |  | F=0 (1, 968) | p=0.940 |  | F=3.6 (1, 968) | p=0.057 |
| Mental health diagnosis | F=1.8 (1,959) | p=0.182 |  | F=0.2 (1, 966) | p=0.672 |  | F=2.1 (1, 968) | p=0.149 |
| Disability diagnoses | F=1.1 (1,968) | p=0.292 |  | F=17,2 (1, 968) | p<0.001* |  | F=1.9 (1, 968) | p=0.171 |
| Daily screen time | F=2.7 (1,968) | p=0.098 |  | F=24.5 (1, 968) | p<0.001* |  | F=16.8 (1, 968) | p<0.001* |
| Household makeup | F=0.7 (3, 967) | p=0.531 |  | F=3.0 (3, 967) | p=0.031 |  | F=0.8 (3, 968) | p=0.488 |
| School location | F=1.6 (1, 19) | p=0.215 |  | F=1.5 (1, 23) | p=0.241 |  | F=10.0 (1, 968) | p=0.002* |
| School sector | F=0.1 (1, 16) | p=0.784 |  | F=0 (1, 16) | p=0.999 |  | F=2.9 (1, 968) | p=0.087 |
| Study group allocation | F=1,4 (1, 21) | p=0.253 |  | F=0 (1, 23) | p=0.951 |  | F=0.1 (1, 968) | p=0.702 |
| Extroversion | F=33.1 (1, 967) | P<0.001* |  | F=23.9 (1, 968) | p<0.001* |  | F=9.5 (1, 968) | p=0.002* |
| Sleep quality | F=96.7 (1, 968) | P<0.001* |  | F=102.9 (1, 967) | p<0.001* |  | F=44.9 (1, 968) | p<0.001* |
| Supportive interactions with friends | F=11.7 (1, 968) | P<0.001* |  | F=5.9 (1, 967) | p=0.015* |  | F=54.3 (1, 968) | p<0.001* |
| Negative interactions with friends | F=1.1 (1, 964) | P=0.298 |  | F=9.7 (1, 963) | p=0.002* |  | F=1.7 (1, 968) | p=0.187 |
| Supportive interactions with family | F=1.7 (1, 968) | P=0.196 |  | F=6.9 (1, 968) | p=0.009* |  | F=15.8 (1, 968) | p<0.001* |
| Negative interactions with family | F=6.0 (1, 967) | P=0.015* |  | F=26.6 (1, 966) | p<0.001* |  | F=6.6 (1, 968) | p=0.010* |
| School connectedness | F=153.4 (1, 964) | P<0.001* |  | F=8.0 (1, 962) | p=0.005* |  | F=62.3 (1, 968) | p<0.001* |
| ICSEA | F=0.7 (1, 16) | P=0.401 |  | F=1.9 (1, 17) | p=0.182 |  | F=7.5 (1, 968) | p=0.006* |
| Linguistic diversity at school-LGA | F=1.0 (1, 20) | P=0.326 |  | F=1.6 (1, 18) | p=0.222 |  | F=0.7 (1, 968) | p=0.392 |
| Country of birth at school-LGA | F=1.2 (1, 20) | P=0.296 |  | F=2.8 (1, 19) | p=0.109 |  | F=0.1 (1, 968) | p=0.731 |

*Note:* Significant p values (p<0.017) are in indicated with *
